# Mapping the Homodimer Interface of an Optimized, Artificial, Transmembrane Protein Activator of the Human Erythropoietin Receptor

**DOI:** 10.1371/journal.pone.0095593

**Published:** 2014-04-30

**Authors:** Emily B. Cohen, Susan J. Jun, Zachary Bears, Francisco N. Barrera, Miriam Alonso, Donald M. Engelman, Daniel DiMaio

**Affiliations:** 1 Department of Genetics, Yale School of Medicine, New Haven, Connecticut, United States of America; 2 Department of Molecular Biophysics & Biochemistry, Yale School of Medicine, New Haven, Connecticut, United States of America; 3 Yale Cancer Center, New Haven, Connecticut, United States of America; 4 Department of Therapeutic Radiology, Yale School of Medicine, New Haven, Connecticut, United States of America; 5 Department of Biochemistry and Cellular and Molecular Biology, University of Tennessee, Knoxville, Knoxville, Tennessee, United States of America; University of Nebraska Medical Center, United States of America

## Abstract

Transmembrane proteins constitute a large fraction of cellular proteins, and specific interactions involving membrane-spanning protein segments play an important role in protein oligomerization, folding, and function. We previously isolated an artificial, dimeric, 44-amino acid transmembrane protein that activates the human erythropoietin receptor (hEPOR) *in trans.* This artificial protein supports limited erythroid differentiation of primary human hematopoietic progenitor cells *in vitro*, even though it does not resemble erythropoietin, the natural ligand of this receptor. Here, we used a directed-evolution approach to explore the structural basis for the ability of transmembrane proteins to activate the hEPOR. A library that expresses thousands of mutants of the transmembrane activator was screened for variants that were more active than the original isolate at inducing growth factor independence in mouse cells expressing the hEPOR. The most active mutant, EBC5-16, supports erythroid differentiation in human cells with activity approaching that of EPO, as assessed by cell-surface expression of glycophorin A, a late-stage marker of erythroid differentiation. EBC5-16 contains a single isoleucine to serine substitution at position 25, which increases its ability to form dimers. Genetic studies confirmed the importance of dimerization for activity and identified the residues constituting the homodimer interface of EBC5-16. The interface requires a GxxxG dimer packing motif and a small amino acid at position 25 for maximal activity, implying that tight packing of the EBC5-16 dimer is a crucial determinant of activity. These experiments identified an artificial protein that causes robust activation of its target in a natural host cell, demonstrated the importance of dimerization of this protein for engagement of the hEPOR, and provided the framework for future structure-function studies of this novel mechanism of receptor activation.

## Introduction

Transmembrane proteins comprise approximately 30% of all cellular proteins [1] and play critical roles in many biological processes. Most membrane-spanning protein segments are hydrophobic α-helical structures, whose transmembrane stability is largely independent of their amino acid sequence. Nevertheless, the sequence of transmembrane domains confers specificity on these protein segments because the amino acid side-chains can engage in highly specific protein-protein interactions in the membrane, which determine protein oligomerization, folding, and activity. It is therefore important to understand the molecular basis for specific protein-protein interactions between transmembrane domains.

Transmembrane domains can be difficult to study due to their localization in membranes and poor solubility in aqueous environments. We have developed genetic methods to circumvent some of the challenges posed by transmembrane domains and used these methods to isolate small, artificial transmembrane proteins that modulate native cellular transmembrane proteins in living cells [2]. Using the dimeric 44-amino acid bovine papillomavirus E5 oncoprotein as a scaffold, we have generated libraries expressing hundreds of thousands of artificial proteins with randomized transmembrane domains and selected biologically active proteins from these libraries. Because the E5 protein is essentially an isolated transmembrane domain, it is an ideal scaffold for constructing such transmembrane protein libraries. Previously, we used this approach to isolate small transmembrane proteins that activate the natural cellular target of the E5 protein, the platelet-derived growth factor beta receptor (PDGFβR) [3]–[6]. We also isolated small transmembrane proteins that activate the human erythropoietin receptor (hEPOR) [7] or down-regulate CCR5 [8], a multi-pass transmembrane G protein-coupled receptor and HIV entry co-receptor. Our success in reprogramming E5 to recognize completely different targets highlights the ability of transmembrane domains to engage in highly specific inter-helical interactions that can modulate complex biological processes [9]–[11]. We designate these small transmembrane proteins “traptamers,” for transmembrane aptamers.

**Figure 1:**
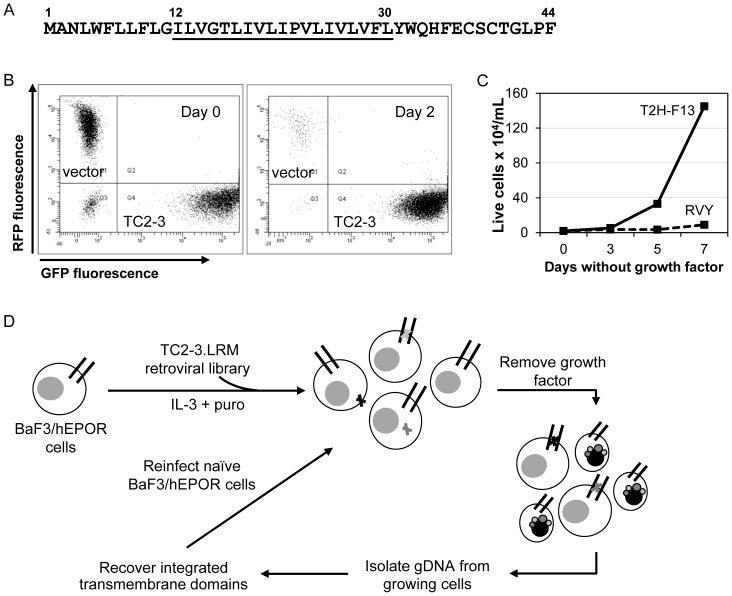
TC2-3 confers cell-autonomous, dose-dependent growth factor independence in hEPOR cells. (**A**) The sequence of TC2-3, which was used as a template to generate a retrovirus expression library in which a 19-amino acid transmembrane segment (positions 12 to 30, underlined) was mutagenized. All other residues are derived from the E5 protein and remained unchanged. (**B**) Equal numbers of BaF3/hEPOR cells expressing RFP alone (vector) or co-expressing TC2-3 and GFP (TC2-3) were co-cultured. Viable cells were analyzed by flow cytometry for GFP and RFP fluorescence immediately after mixing (left panel) and after two days in the absence of growth factors (right panel). (**C**) BaF3/hEPOR cells were infected with retrovirus expressing TC2-3 from a low expression vector, RVY-hygro (dashed line), or a high expression vector, T2H-F13 (solid line). After selection with hygromycin, viable cells were counted on the indicated days after growth factor removal. (**D**) Scheme to select optimized small transmembrane activators of the hEPOR. Black lines represent the hEPOR and gray and black X’s represent small transmembrane proteins. Small cells with nuclear blebs represent dead cells.

EPO normally functions by activating the EPOR, a single-pass transmembrane cytokine receptor required for erythroid differentiation and red blood cell production. TC2-3, the traptamer that activates the hEPOR, supports limited erythroid differentiation in primary human hematopoietic progenitor cells (hHPCs) *in vitro* in the absence of EPO [7]. TC2-3 consists of a 19-amino acid random transmembrane segment flanked by 25 amino acids from E5 (Fig. 1A), forms a homodimer, and displays no sequence or biochemical similarity to EPO. TC2-3 does not activate the PDGFβR or the murine EPOR, and the transmembrane domain of the hEPOR is required for TC2-3 action [7]. We reasoned that isolation of a more active mutant of TC2-3 would facilitate the analysis of small transmembrane activators of the hEPOR and allow the identification of specific features of these proteins that are important for their activity.

Here, we used a directed evolution approach to isolate a mutant of TC2-3 with increased activity. A library encoding thousands of TC2-3 mutants was subjected to selection under stringent conditions to isolate a traptamer with enhanced activity, EBC5-16, which contains a single amino acid substitution that increases dimerization. When expressed in hHPCs, EBC5-16 induces cell-surface expression of the erythroid-specific, differentiation marker, glycophorin A (GpA), to the same extent as in cells stimulated with EPO. These results suggest that dimerization of EBC5-16 plays a key role in its ability to induce erythroid differentiation. As a first step in understanding the molecular basis for the activity of EBC5-16, we conducted genetic analysis to identify and characterize its homodimer interface. These experiments provide evidence that increased dimerization of EBC5-16 is responsible for its enhanced activity. This work represents a novel approach to isolate and characterize potent, specific, biologically active proteins not found in nature, which have the potential to modulate the activity of a diverse array of cellular transmembrane proteins of research and clinical importance. In addition, study of these proteins will provide insight into protein-protein interactions occurring in membranes.

## Materials and Methods

### Ethics Statement

Human Subjects: All work was conducted according to Declaration of Helsinki principles. Collection and use of human cells was approved by the Yale University institutional review board. Written informed consent was received from participants prior to use of their extra G-CSF mobilized cells in the study. (HIC protocol #0309025874, Voluntary Donation of Excess Peripheral Mononuclear Cells Collected via Apherisis for Research on Stem Cells. Approved 10/26/11. Principal Investigator: Krause, Diane S.)

### Plasmids and Cloning

The TC2-3 limited random mutagenesis library (described below) was cloned into a modified pT2H-F13 vector (details of construction of original vector described in Cammett *et al.* [7]) without a Kozak consensus sequence, resulting in an alanine to proline mutation at position two of the E5 protein. In addition, the hygromycin resistance gene in the pT2H-F13 was replaced with a puromycin resistance gene. The resulting low expression retroviral vector was named pRVY-puro.

The HA-tagged hEPOR (originally obtained from S. Constantinescu) and HA-hEPOR(mPR) (described in Cammett *et al.* [7]) genes were excised from the pBABE-puro retroviral vector and cloned into the high expression vector, pMSCV-neo (Clontech), using standard cloning techniques. EBC5-16 was subcloned into pCMMP-IRES-GFP (gift from B. Sugden, University of Wisconsin) using standard cloning techniques (as described in Cammett *et al.* [7]).

The Put3/5-16 chimeras were generated by using splice-overlap PCR and Pfu Turbo polymerase (Agilent) (as described in Mattoon *et al.* [12] with modifications; details of this and other cloning procedures are available from the authors upon request). The resulting fragments were PCR purified, digested with XhoI and BamHI, and cloned into pRVY-puro. The DNA sequence of each chimeric Put3/5-16 gene was confirmed. The chimeras are numbered (in roman numerals) according to the number of codons inserted at the point of fusion.

The double cysteine-to-serine mutation in EBC5-16 (EBC5-16-CCSS) was generated by using double-stranded oligonucleotides, which were cloned into EBC5-16 in pMSCV-puro. Point mutations in EBC5-16 were generated by using Quick Change (Agilent) site-directed mutagenesis using codon-optimized EBC5-16 cloned into the retroviral vector, pMSCV-puro (Clontech), as the starting template. pRVY-hygro/TC2-3 and pRVY-hygro/EBC5-16 were generated by cloning TC2-3 and EBC5-16, respectively, from pRVY-puro to pRVY-hygro using standard cloning techniques. pRVY-hygro/EBC5-16 S25A was generated by using Quick Change site-directed mutagenesis using pRVY-hygro/EBC5-16 as the starting template. The poly-leucine gene and add-back constructs were generated using complementary oligonucleotides, which were cloned into pMSCV-puro. Oligonucleotides used here are listed in Table S1.

### Cells, Viruses, and Tissue Culture

293T cells were maintained in Dulbecco’s Modified Eagle Medium (DMEM) supplemented with 5% fetal bovine serum (FBS) (Gemini Bioproducts) and 5% bovine calf serum (BCS) (Gemini Bioproducts), 4 mM L-glutamine, 20 mM HEPES (pH 7.3), and 1X penicillin/streptomycin (P-S) (DMEM-10). Murine interleukin-3 (IL-3)-dependent BaF3 cells were maintained in RPMI-1640 supplemented with 10% heat-inactivated FBS, 5% WEHI-3B cell-conditioned medium (as the source of IL-3), 2 mM L-glutamine, 0.06 mM β-mercaptoethanol, and 1X P-S (RPMI-IL-3).

Vesicular stomatitis virus (VSV)-G protein pseudotyped retroviruses were prepared by using calcium phosphate precipitation to co-transfect 293T cells with a retroviral plasmid and pantropic VSVg (Clontech) and pCL-Eco (Imgenex) retroviral packaging plasmids [13]. After culture in DMEM-10 or OptiMEM Reduced Serum Medium (Gibco) for 48 hours at 37°C, the viral supernatant was harvested, filtered through a 0.45 µm filter (Millipore), and either used immediately or concentrated approximately 20X by using Amicon Ultra-15 columns, Centricon Ultracel PL-30 (Millipore), or PEG-it Virus Precipitation Solution (System Biosciences).

BaF3 cells expressing untagged hEPOR and mPDGFR from pBABE-puro were previously described [6], [7]. BaF3 cells expressing HA-tagged hEPOR (HA-hEPOR) and mEPOR (HA-mEPOR) were generated by infecting BaF3 cells in RPMI-IL-3 with retrovirus expression vector MSCV-neo/HA-hEPOR and MSCV-neo/HA-mEPOR, respectively, followed by selection with 1 mg/mL G418. Cells were then washed with phosphate buffered saline (PBS) and resuspended in RPMI medium lacking IL-3 but containing 0.5 U/mL Epogen (Epoietin Alfa, Amgen) recombinant EPO (RPMI-EPO). A clonal cell line expressing HA-tagged human EPOR (BaF3/HA-hEPOR) was established by serial dilution in RPMI-EPO.

### Cell-autonomy Assay

BaF3/hEPOR cells in RPMI-IL-3 were infected with concentrated stocks of CMMP-IRES-RFP or CMMP-IRES-GFP/TC2-3. One hundred thousand BaF3/hEPOR/CMMP-IRES-RFP cells were then co-cultured with 1×10^5^ BaF3/hEPOR/CMMP-IRES-GFP/TC2-3 cells, washed once with PBS, and resuspended in RPMI medium lacking IL-3 and EPO. At various time points, RFP and GFP fluorescence were analyzed by flow cytometry on a BD LSRII Green at 488 and 532 nm.

### Limited Random Mutagenesis Library Construction

The library expressing randomized mutants of TC2-3 was constructed using a degenerate oligonucleotide mixture in which codons 12 to 30 of TC2-3 were mutagenized, while the remaining codons, 1 to 11 and 31 to 44, remained fixed as TC2-3 sequences. To allow an average of two to three amino substitutions per transmembrane domain, the ratio of nucleotides at each mutagenized position was 94% of the wild-type nucleotide from TC2-3 and 2% each of the other three nucleotides. The degenerate oligonucleotide was annealed to a non-degenerate oligonucleotide, which was complementary to the 3’ fixed sequences of the degenerate oligonucleotide and encoded a stop codon and a BamHI restriction site. The oligonucleotides were annealed, extended, and amplified by PCR using short primers complementary to the fixed sequences at the ends of the long oligonucleotides, digested with AvrII and BamHI, and cloned as a mixture of fragments into pRVY-puro. The ligation reaction was used to transform *E. coli* strain DH10β (Invitrogen). Colonies were picked at random and sequenced to confirm the composition of the library. Lawns of ∼1.6×10^6^ transformed bacterial colonies were pooled, and plasmid DNA was harvested from this pool and named pRVY-TC2-3 limited random mutagenesis (LRM) library (TC2-3.LRM). Oligonucleotides used for library construction, recovery, and mutagenesis are listed in Table S1.

### Library Infection and Genetic Selection of Growth Factor-Independent Cells

Five wells of 5×10^5^ BaF3/HA-hEPOR cells were plated in a 12-well plate in 500 µl of RPMI-IL-3. Five hundred µl of 20X concentrated TC2-3.LRM virus was added to each well. Polybrene was added to a final concentration of 4 µg/mL. Cells were incubated for four hours and then transferred to individual 25 cm^2^ flasks (Corning) containing 9 mL of RPMI-IL-3 with polybrene. Two days post-infection, 1 µg/mL puromycin was added to each flask. Four days post-infection, when mock-infected cultures were dead, 5×10^5^ cells from each flask were washed twice in PBS and resuspended in 10 mL RPMI lacking IL-3 and EPO [RPMI-no growth factor (noGF)]. After eight days of selection, cells were harvested from each pool, genomic DNA was isolated (DNeasy, Qiagen), and inserts recovered by PCR (Expand Long Template PCR kit, Roche) using primers that annealed to the fixed regions of the TC2-3 gene (primers listed in Table S1). The PCR products were purified, digested with AvrII and BamHI, cloned into pRVY-puro, and packaged into retrovirus to generate secondary libraries.

Each secondary library was separately packaged into retrovirus, concentrated, and used to infect two wells of naïve BaF3/HA-hEPOR cells as described above. Two days post-infection, puromycin was added to each flask at a final concentration of 1 µg/mL. Four days post-infection, 5×10^5^ cells from each flask were harvested, washed twice in PBS, and transferred to 10 mL RPMI-noGF. Eight days after selection, cells were harvested and genomic DNA was isolated. Inserts were recovered by PCR, cloned into pRVY-puro, and sequenced. Clones recovered from this selection were packaged individually into retrovirus and used to infect BaF3/HA-hEPOR cells. For each infection, approximately 3×10^5^ cells/well in 200 µl RPMI-IL-3 were infected with 1 mL unconcentrated, freshly prepared retrovirus as described above. After selection in puromycin, 2×10^5^ viable cells of each infection were washed once and resuspended in RPMI-noGF. Viable cells were counted every two days using an Invitrogen Countess Cell Counter.

### Immunoprecipitation and Immunoblotting

For HA-hEPOR phosphotyrosine blotting, cells were cytokine-starved overnight and, in some cases, acutely stimulated with 5 U/mL EPO for 5 min at 37°C [7]. Cells were then washed twice with PBS containing 1 mM phenylmethylsulfonyl fluoride (PMSF) and 500 µM H_2_O_2_-activated sodium metavanadate and lysed in RIPA-MOPS (20 mM morpholinepropanesulfonic acid [pH 7.0], 150 mM NaCl, 1 mM EDTA, 1% Nonidet P-40, 1% deoxycholate, and 1% SDS) buffer containing protease and phosphatase inhibitors (HALT Protease and Phosphatase Inhibitor Cocktail, Thermo Scientific), 1 mM PMSF, and 500 µM H_2_O_2_-activated sodium metavanadate. To immunoprecipitate HA-hEPOR, 50 µl of Roche anti-HA affinity matrix (immobilized rat monoclonal, Clone 3F10) was added to 1 mg of extracted protein and rotated overnight at 4°C. For blotting of phosphorylated JAK2 and phosphorylated STAT5, cells were cytokine-starved overnight and, in some cases, acutely stimulated with 5 U/mL EPO or RPMI-IL-3 for 5 min at 37°C. To immunoprecipitate TC2-3, EBC5-16, or an EBC5-16 mutant or fusion protein, 10 µl of a rabbit polyclonal antibody against the fixed 16 C-terminal residues of the E5 protein was added to 1 mg of RIPA-MOPS protein lysate and rotated overnight at 4°C, and 50 µl Protein A Sepharose bead slurry was added for two hours at 4°C.

Immunoprecipitated samples were washed three times with NETN buffer (100 mM NaCl, 0.1 mM EDTA, 20 mM Tris-HCl [pH 8.0], 0.1% Nonidet P-40) supplemented with 1 mM PMSF (for phosphotyrosine and phospho-protein blots, 500 µM H_2_O_2_-activated sodium metavanadate was also present during washing), pelleted and resuspended in 2x Laemmli sample buffer with or without 200 mM DTT and 5% β-mercaptoethanol. Precipitated proteins and whole cell lysates were resolved by SDS-PAGE on a 7.5% polyacrylamide gel for total and phosphorylated JAK2 blotting, 10% polyacrylamide for HA-hEPOR, phosphotyrosine, and total and phosphorylated STAT5 blotting, or a 20% polyacrylamide gel for E5 blotting. The resolving gel was transferred to a 0.45 µm polyvinylidene fluoride (PVDF) membrane for HA-hEPOR, 0.45 µm nitrocellulose for phosphotyrosine blotting, or 0.2 µm PVDF membrane for total and phosphorylated JAK2 and STAT5, and E5 blotting using standard procedures (gels for E5 blotting were transferred without SDS).

Membranes were blocked with gentle agitation for one hour at room temperature in 5% bovine serum albumin (BSA) in 1X Tris buffered saline plus 0.1% Tween-20 (TBST) for phosphotyrosine and for phosphorylated and total JAK2 blots. 5% nonfat dry milk/TBST was used for all other blots. Mouse anti-phosphotyrosine monoclonal antibody PY100 (Cell Signaling) was used at a 1∶2000 dilution in 5% BSA/TBST to detect phosphorylated EPOR, a 1∶500 dilution of a rabbit anti-EPOR antibody (clone C-20, Santa Cruz Biotechnology) was used in 5% milk/TBST to detect HA-hEPOR, a 1∶1000 dilution of rabbit anti-JAK2 monoclonal antibody (clone D2E12, Cell Signaling) in 5% BSA/TBST was used to detect JAK2, a 1∶1000 dilution of a rabbit anti-phospho-JAK2 monoclonal antibody (Tyr1008) (clone D4A8, Cell Signaling) in 5% BSA/TBST was used to detect phosphorylated JAK2, a 1∶1000 dilution of a rabbit anti-STAT5b antibody (Chemicon) in 5% milk/TBST was used to detect STAT5, a 1∶1000 dilution of a mouse anti-phospho-STAT5 monoclonal antibody (Tyr694) (clone 14H2, Cell Signaling) in 5% milk/TBST was used to detect phosphorylated STAT5, a 1∶250 dilution of a rabbit anti-E5 polyclonal antibody to detect TC2-3, EBC5-16, and EBC5-16 mutants, and a 1∶1000 dilution of a mouse anti-AU1 monoclonal antibody (Abcam) was used to detect Put3/EBC5-16 fusion proteins. All membranes were incubated overnight with gentle agitation in primary antibody at 4°C, washed five times in TBST, and then incubated with gentle agitation for one hour at room temperature in donkey anti-mouse or anti-rabbit HRP (Jackson Immunoresearch), as appropriate, at a 1∶10,000 dilution or in Protein A HRP (Amersham or Pierce) for polyclonal rabbit antibody blots at a 1∶8000 dilution in blocking buffer. To reprobe phospho-JAK2 and phospho-STAT5 blots, membranes were stripped in Restore Western Stripping Buffer (Thermo Scientific) for 10 min at room temperature with gentle agitation, washed five times in TBST, blocked in 5% BSA/TBST (JAK2) or 5% milk/TBST (STAT5) for one hour at room temperature, and incubated overnight at 4°C with JAK2 or STAT5 antibody as described above. All membranes were incubated with SuperSignal West Pico or Femto Chemiluminescent Substrates (Pierce) to detect protein bands.

### Transduction of Human CD34+ Cells and Erythroid Differentiation Assay

Human CD34^+^ cells were obtained from healthy adult donors by G-CSF-mobilized peripheral blood apheresis, selected by using the Baxter 300i Isolex device, and cryopreserved at -80°C. The cells were cultured for four days in StemSpan Serum-Free Expansion Medium (Stem Cell Technologies) supplemented with 20 ng/mL recombinant human (rh)-IL-6, 100 ng/mL rh-stem cell factor (SCF), 100 ng/mL rh-Flt-3 ligand, and 20 ng/mL IL-3 (StemSpan Cytokine Cocktail, Stem Cell Technologies). Five hundred thousand CD34^+^ cells in 500 µl expansion medium per well of a 12-well plate were infected with 500 µl of concentrated CMMP-IRES-GFP, CMMP-IRES-GFP/TC2-3 or CMMP-IRES-GFP/EBC5-16 by spinoculation (900 rpm for one hour at room temperature) in the presence of 8 µg/mL polybrene. The infected cells were incubated overnight at 37°C and then transferred to a 6-well dish with fresh medium. Forty-eight hours post-infection, GFP-expressing cells were isolated by sterile cell sorting on a BD FACS Vantage SE or Sony SY3200 at 488 nm.

GFP^+^ CD34^+^ cells were seeded at a density of 3×10^5^ cells/mL in differentiation medium: 20 ng/mL rh-SCF (ConnStem), 5 ng/mL rh-IL-3 (ConnStem), 0.2 µM β-Estradiol (Sigma), 2 µM dexamethasone (Sigma) in StemSpan Serum-Free Medium in the absence or presence of 1 U/mL EPO [14]. Viable cells were counted at various days. The cell cultures were diluted over time with fresh medium, as necessary, to maintain the cell concentration at approximately 3×10^5^ cells/mL, and cell counts were corrected for dilution.

After various times in differentiation medium, 1×10^5^ cells were washed once in 0.5% BSA/PBS and incubated with a mouse anti-human glycophorin A (GpA) monoclonal antibody (clone HIR2, eBioscience) on ice for 20 minutes. The cells were then washed twice with 0.5% BSA/PBS, incubated with allophycocyanin-conjugated donkey anti-mouse polyclonal antibody (eBioscience) on ice for 20 minutes in the dark, washed twice in 0.5% BSA/PBS, and analyzed by flow cytometry for cell-surface GpA expression on a BD FACSCalibur at 633 nm.

For quantitative real-time reverse transcriptase PCR (qRT-PCR) analysis of human β-globin transcription, total RNA was isolated from 5×10^5^ GFP-expressing hHPCs grown for 6 days in differentiation medium by using QiaShredder, RNeasy Mini, and RNase-free DNase kits (Qiagen). One µg RNA was used as a template for cDNA synthesis using an iScript synthesis kit (BioRad). Using the BioRad MyiQ Single-color, qRT-PCR was performed with iQ SYBR Green Supermix (BioRad) and 40 ng cDNA per 20 µl reaction. Samples were heated 3 minutes at 95°C and then subjected to 40 cycles of denaturation at 95°C for 30 seconds, annealing at 60°C for 30 seconds, and extension at 60°C for 1 minute. Gene-specific primers were designed using the Universal Probe Library Probe-Finder software (Roche) and are listed in Table S1. qRT-PCR values for β-globin mRNA were normalized to GAPDH mRNA for each sample, followed by normalization to the negative control, CMMP-IRES-GFP without EPO, to determine relative β-globin expression for each sample.

For erythroid colony forming assays in methylcellulose, 1×10^4^ GFP^+^-infected cells/mL were washed in Iscove’s Modified Dulbecco’s Medium (L-glutamine, 25 mM HEPES, 3.024 g/L Na_2_CO_3_) (Gibco) plus 2% FBS (Stem Cell Technologies) and diluted 1∶10 in methylcellulose medium (Methocult H4531, Stem Cell Technologies) containing 20 ng/mL rh-IL-3, 20 ng/mL rh-IL-6 (ConnStem), 50 ng/mL rh-SCF in the presence or absence of 3 U/mL of EPO. One thousand cells were plated per 35 mm dish, and colony formation and benzidine staining were assessed at day 14.

### TOXCAT Assay for Transmembrane Domain Oligomerization

To construct TOXCAT chimeric constructs, the sequence encoding amino acids 8 to 32 of EBC5-16 and TC2-3 was amplified and cloned into the pccKAN vector between the sequences encoding the N-terminal DNA binding domain of ToxR and the maltose binding protein [15]. These fusion proteins (and controls containing the transmembrane domains of GpA, which forms a strong dimer, and a GpA mutant with decreased dimerization (G83I mutant)) were expressed in *E. coli*. The level of oligomerization was measured by quantification of chloramphenical acetyl transferase (CAT) activity using ^3^H-labeled chloramphenicol, as described in Russ and Engelman, 1999 [15]. CAT activity was normalized to the expression level of each chimera as determined by Western blotting with an antibody against the maltose binding protein (ZYMED Laboratories). For each independent experiment, CAT activity was assayed in triplicate.

### Molecular Modeling

Models for transmembrane dimers were generated by using the CHI (Crystallography and NMR system Helical Interactions) computational method [16]. Briefly, CHI was used to construct a symmetric pair of canonical alpha helices. Molecular dynamics (MD) simulations are performed *in vacuo* by using simulated annealing of atomic coordinates. Energy minimization was performed before and after MD simulations, and structures were clustered into groups with a backbone root mean square deviation (RMSD) of 1 Å. This procedure defines basins of convergence for helix pairs having chemically reasonable structures. The search was carried out over the entire symmetric two-body rotational interaction space (0-360°), with an inter-helix distance of 10 Å and a crossing angle of 10°, both typical values for transmembrane helical dimers.

## Results

### Small Transmembrane Activator of the hEPOR Acts in a Cell-Autonomous, Dose-Dependent Fashion

To gain a better understanding of the structure of small transmembrane activators of the hEPOR and facilitate mechanistic studies, we isolated a more active version of TC2-3. To accomplish this, we first determined whether TC2-3 acts in a cell-autonomous fashion or induces the secretion of a soluble factor responsible for growth factor independence. This experiment was conducted in BaF3/hEPOR cells, an IL-3-dependent murine cell line, in which expression of TC2-3 abrogates IL-3 dependence by activating an exogenously expressed hEPOR. A CMMP retrovirus vector with an internal ribosome entry site (IRES) was used to co-express TC2-3 and green fluorescent protein (GFP) from a single transcript in BaF3/hEPOR cells. These cells were co-cultured with an equal number of BaF3/hEPOR cells expressing red fluorescent protein (RFP) but lacking TC2-3. After growth factor removal and further incubation, the proportion of GFP- and RFP-expressing cells in the culture was assessed by flow cytometry. As shown in Figure 1B, at the time of growth factor removal, the GFP and RFP cells were present in equal number. However, within two days of growth factor removal, the vast majority of cells expressing RFP (*i.e.*, those lacking TC2-3 expression) died, whereas the cells expressing TC2-3 and GFP proliferated due to activation of the hEPOR by TC2-3. The relative proportion of GFP^+^ cells in the population increased with extended incubation times in the absence of growth factors (data not shown). Thus, cells expressing TC2-3 do not secrete a factor that stimulates growth of BaF3/hEPOR cells lacking TC2-3 in the same culture, demonstrating that TC2-3 activates the hEPOR in a cell-autonomous manner. Because of this property, we were able to use a genetic method to screen a large number of TC2-3 mutants in mixed culture for those with increased activity, because the effect of each mutant is restricted to the cell expressing it, thereby allowing us to isolate rare active clones.

We also needed a system in which TC2-3 was minimally active, so that a more active version would confer a selectable phenotype. BaF3/hEPOR cells grow robustly in the absence of growth factors if TC2-3 was expressed from a high expression vector, such as T2H-F13, but low-level expression of TC2-3 from the RVY-hygro vector supports minimal growth factor-independent proliferation (Fig. 1C). Thus, TC2-3 mutants that induced growth factor independence when expressed at a low level in BaF3/hEPOR cells were likely to be more active than TC2-3 itself.

### Isolation and Characterization of a More Potent Transmembrane Activator of the hEPOR

To isolate TC2-3 mutants with enhanced activity, we subjected the transmembrane domain of TC2-3 (amino acid positions 12 to 30) to limited random mutagenesis (Fig. 1A). We used a degenerate oligonucleotide in which each position encoding the transmembrane segment was synthesized with a nucleotide mixture consisting of the wild-type nucleotide “doped” with a low percentage of each non-wild-type nucleotide. This oligonucleotide was converted into double-stranded DNA, amplified, and cloned into the low expression vector, pRVY-puro, to generate a library named TC2-3.LRM, which encodes an estimated 15,000 different TC2-3 mutants with an average of two to three amino acid substitutions per protein.

**Figure 2:**
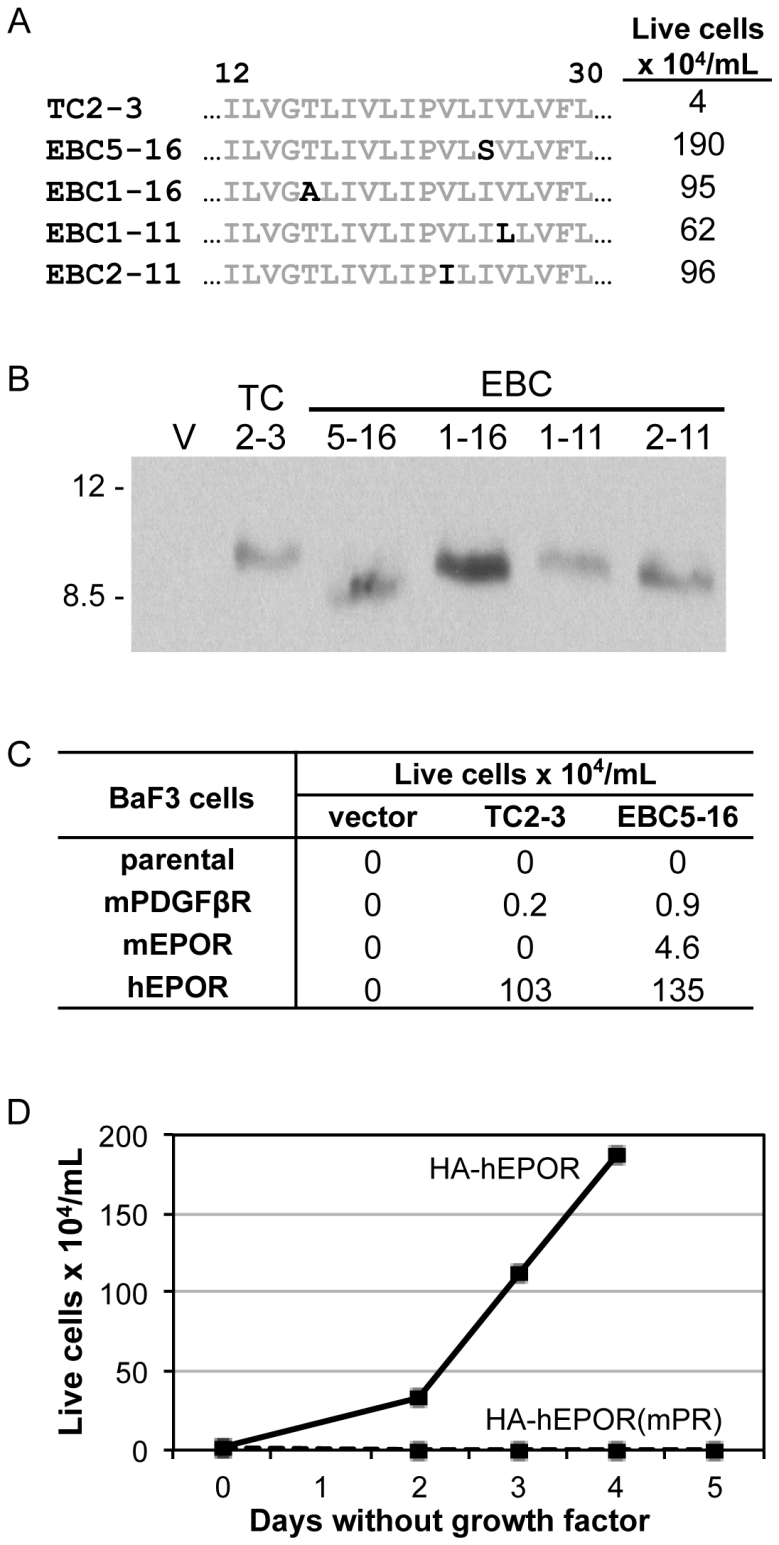
Transmembrane protein mutants with single amino acid substitutions display increased activity compared to TC2-3. (**A**) (Left) Amino acid sequence of the transmembrane domain (positions 12 to 30) of TC2-3 and the mutants selected from the library. Residues in black indicate amino acid substitutions. (Right) BaF3/HA-hEPOR cells expressing TC2-3 or the selected mutants expressed from the low expression vector, RVY-puro, were tested for their ability to proliferate in the absence of growth factors. Viable cells were counted four days after growth factor removal. (**B**) Extracts were prepared from BaF3/HA-hEPOR cells expressing empty RVY-puro vector, TC2-3, or the indicated mutant. Samples were immunoprecipitated and immunoblotted with αE5. Size of protein markers (in kDa) is shown on left. (**C**) Empty MSCV-puro vector, TC2-3, or EBC5-16 were expressed in BaF3 cells expressing no exogenous receptor, murine PDGFβR, murine EPOR, or hEPOR. Cells were then tested for their ability to proliferate in the absence of growth factors. Viable cells were counted three days after growth factor removal. TC2-3 was active with hEPOR in this experiment because it was expressed from MSCV. (**D**) MSCV-puro/EBC5-16 was expressed in BaF3 cells expressing either HA-hEPOR (solid line) or HA-hEPOR(mPR) (dashed line), and cells were tested for their ability to proliferate in the absence of growth factors. Viable cells were counted on the indicated days.

Figure 1D shows the strategy used to isolate mutants of TC2-3 with increased activity. We infected several pools of BaF3/HA-hEPOR cells with the TC2-3.LRM library at a low multiplicity of infection (MOI), selected with puromycin for stable transduction of the mutant TC2-3 genes, and incubated transduced cells in the absence of growth factors. After eight days, cells infected with the library proliferated robustly in the absence of growth factors, but cells infected with the empty RVY-puro vector did not. The library inserts from the genomic DNA of these growth factor-independent cells were amplified, cloned as pools into pRVY-puro, and packaged *en masse* to generate individual secondary libraries. After infecting naïve BaF3/HA-hEPOR cells with each secondary library and repeating the selection for growth factor independence, a number of TC2-3 mutants were recovered from proliferating cells. Each of these mutants contains a single amino acid substitution at a different position in the mutagenized transmembrane segment (Fig. 2A). These mutants were expressed individually from RVY-puro in BaF3/HA-hEPOR cells and tested for their ability to confer growth factor independence. Several of these TC2-3 mutants were more active than TC2-3 in this assay (Fig. 2A). Immunoprecipitation and Western blotting revealed that most of these TC2-3 mutants were not expressed at higher levels than TC2-3 itself (Fig. 2B), so their increased activity is not simply a consequence of increased expression. One mutant, designated EBC5-16, contains an isoleucine to serine mutation at position 25 and was reproducibly the most active in conferring growth factor independence. Inserting any of the other mutations identified in the screen into EBC5-16 did not further enhance its activity (data not shown), so we focused on EBC5-16 itself for further experiments. For comparisons between TC2-3 and EBC5-16 and between the Put3/EBC5-16 chimeras (see below), we typically used the RVY low expression vector (except in hHPCs, where we used the CMMP IRES-GFP vector). In most other experiments (*e.g.*, analysis of EBC5-16 mutants or the ability of EBC5-16 to activate receptor mutants), we used the higher expression vector MSCV to obtain more robust activity.

When EBC5-16 was expressed from the high expression vector, MSCV, it did not confer growth factor independence in parental BaF3 cells lacking hEPOR expression, and like TC2-3 itself [7] displayed minimal activity in BaF3 cells expressing the murine EPOR or PDGFβR (Fig. 2C). Thus, the activity of EBC5-16 is dependent on expression of the EPOR and specific for the human as opposed to the murine version of the receptor. To test whether the transmembrane domain of the hEPOR is required for EBC5-16 activity, we introduced EBC5-16 into cells expressing an HA-tagged hEPOR mutant in which the transmembrane domain of the hEPOR was replaced with that of the murine PDGFβR (designated HA-hEPOR(mPR)). We previously showed that BaF3 cells expressing HA-hEPOR(mPR) proliferated in response to EPO, which binds to the extracellular domain of the receptor retained in the chimera, but did not respond to TC2-3 because of the foreign transmembrane domain [7]. As shown in Figure 2D, EBC5-16 also failed to cooperate with HA-hEPOR(mPR) to induce growth factor independence, indicating that EBC5-16 requires the hEPOR transmembrane domain for activity.

**Figure 3:**
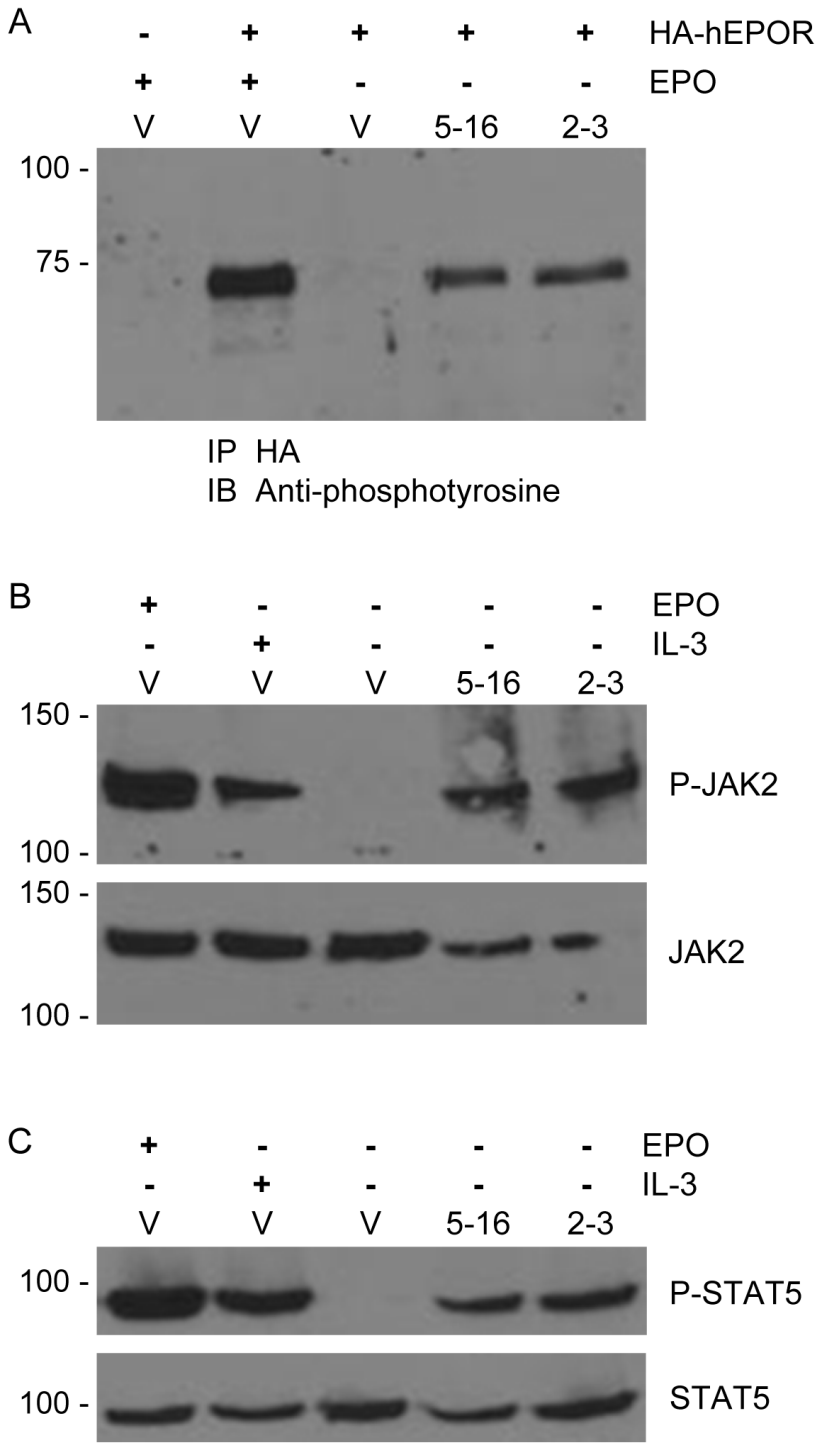
EBC5-16 induces tyrosine phosphorylation of hEPOR, JAK2, and STAT5. (**A**) Extracts were prepared from parental BaF3 cells or BaF3/HA-hEPOR cells expressing empty RVY-puro vector (V), EBC5-16, or TC2-3. Where indicated, cells were acutely stimulated with EPO. Samples were immunoprecipitated with anti-HA (3F10) antibody and immunoblotted with anti-phosphotyrosine antibody. Size of protein markers (in kDa) is shown on left. (**B**) Extracts from BaF3/HA-hEPOR cells expressing RVY-puro vector (V), EBC5-16, or TC2-3. Where indicated, cells were acutely stimulated with EPO or RPMI-IL-3 medium. Samples were immunoblotted for phosphorylated JAK2. Blot was reprobed for total JAK2. Size of protein markers (in kDa) is shown on left. (**C**) Extracts from the cells described in (**B**) were immunoblotted for phosphorylated STAT5. Blot was reprobed for total STAT5. Size of protein markers (in kDa) is shown on left.

To determine whether EBC5-16 causes biochemical activation of the hEPOR, we immunoprecipitated the HA-tagged hEPOR from BaF3/HA-hEPOR cells expressing EBC5-16 or TC2-3 from the low expression vector, RVY-puro, and immunoblotted with an anti-phosphotyrosine antibody. As shown in Figure 3A, EBC5-16 induced tyrosine phosphorylation of the hEPOR. Interestingly, EBC5-16 and TC2-3 induced a similar level of hEPOR tyrosine phosphorylation, despite the enhanced biological activity of EBC5-16. Similarly, EBC5-16 induced tyrosine phosphorylation of JAK2 and STAT5 (Figs. 3B and 3C), major downstream signaling partners of the hEPOR, at levels similar to that induced by TC2-3. These experiments demonstrated that we have isolated a TC2-3 mutant with enhanced biological activity in murine cells, but the basis for enhanced signaling has yet to be determined.

### EBC5-16 Displays Increased Activity in Human Hematopoietic Progenitor Cells

**Figure 4:**
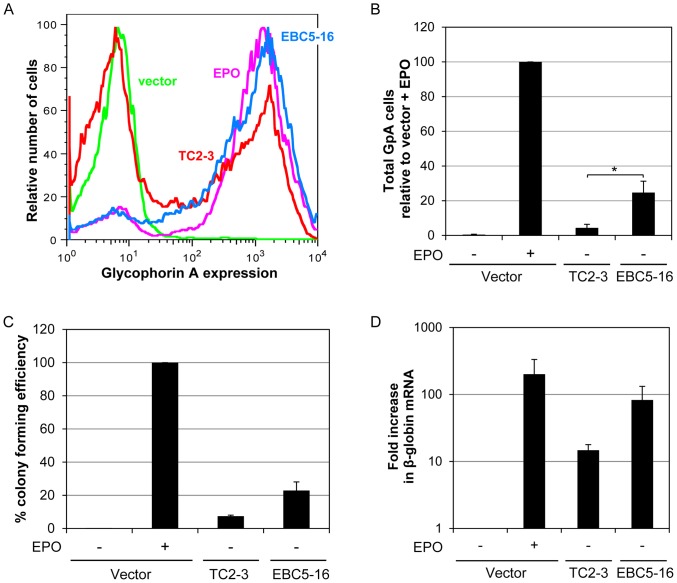
EBC5-16 displays increased ability to stimulate erythroid differentiation of human hematopoietic progenitor cells. (A) Primary human CD34^+^ cells infected with retrovirus expressing empty CMMP-IRES-GFP vector (green), or CMMP-IRES-GFP expressing TC2-3 (red) or EBC5-16 (blue) were sorted for GFP fluorescence and transferred to differentiation medium in the absence of EPO. A sample of cells expressing vector was also treated with EPO (magenta). After six days in differentiation medium, viable cells were assessed for cell-surface GpA expression by immunostaining and flow cytometry. Similar results were obtained in four independent experiments. (B) Cells were handled as in (A). After six days in differentiation medium, the total number of viable cells expressing GpA (>50 fluorescence units) was determined by immunostaining and flow cytometry. Graph shows average of three independent experiments. Error bars represent the standard error of the mean. A student t-test determined the difference between EBC5-16 and TC2-3 samples to be statistically significant, p<0.05. (C) Cells handled as in (A), but cultured in differentiation medium in methylcellulose to measure erythroid colony formation. EPO was added where indicated. Percent colony forming efficiency is relative to vector plus EPO. Graph shows the average of three independent experiments. Error bars represent the standard error of the mean. A student t-test determined the difference between EBC5-16 and TC2-3 samples not to be statistically significant. (D) After six days in differentiation medium, total RNA was isolated from hHPCs expressing empty vector, TC2-3, or EBC5-16. EPO was added where indicated. Levels of human β-globin mRNA were determined by qRT-PCR relative to GAPDH mRNA. Expression is normalized to vector-infected cells in the absence of EPO. Graph shows average of three independent experiments. Error bars represent the standard error of the mean. A student t-test determined the difference between EBC5-16 and TC2-3 samples not to be statistically significant.

To test the activity of EBC5-16 in hHPCs, we cloned it into the pCMMP-IRES-GFP vector, which also encodes GFP from an IRES. Primary CD34^+^ hHPCs were infected with empty CMMP, CMMP expressing TC2-3, or CMMP expressing EBC5-16, and transduced cells were isolated by sorting for GFP fluorescence. Cells were then incubated in serum-free differentiation medium, and several markers of erythroid differentiation were assessed. As expected, hHPCs infected with empty CMMP and incubated in the absence of EPO did not express cell-surface GpA, whereas virtually all cells infected with the empty vector and treated with EPO expressed high levels of cell-surface GpA (Fig. 4A). As previously reported, approximately 50% of the cells transduced with TC2-3 expressed cell-surface GpA in the absence of EPO [7]. Strikingly, more than 90% of the cells infected with the virus expressing EBC5-16 expressed high levels of cell-surface GpA in the absence of EPO, comparable to vector-infected EPO-treated cells (Fig. 4A). In addition to the increased fraction of cells expressing GpA, we also observed a statistically-significant increase in the total number of GpA^+^ cells in response to EBC5-16 compared to TC2-3 (Fig. 4B). When assessed for erythroid colony formation in methylcellulose in the absence of EPO, cells infected with EBC5-16 reproducibly formed more colonies than cells expressing TC2-3, although this difference did not reach statistical significance (Fig. 4C). Similarly, as assessed by qRT-PCR, EBC5-16 reproducibly induced five to ten-fold more β-globin mRNA in hHPCs than TC2-3 (Fig. 4D), although the difference was also not statistically significant. These results demonstrated that the single isoleucine to serine mutation in the transmembrane domain of EBC5-16 renders it more active than TC2-3 in promoting erythroid differentiation, as assessed by several measures of this process.

### Serine at Position 25 Increases Dimerization of EBC5-16

**Figure 5:**
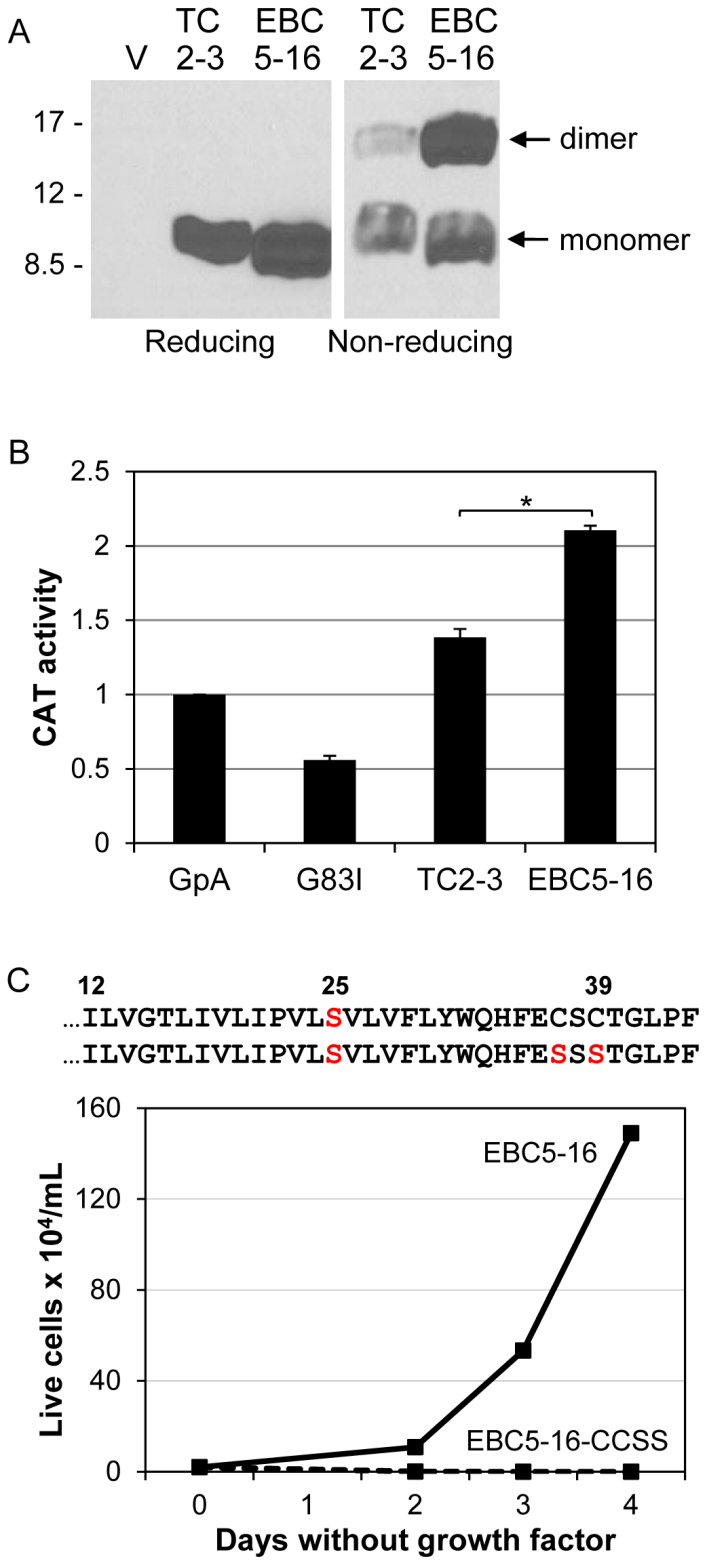
Ser25 increases the formation of EBC5-16 homodimers. (**A**) Extracts were prepared from BaF3/HA-hEPOR cells expressing empty MSCV-puro vector (V), TC2-3, or EBC5-16. Samples were immunoprecipitated with αE5, electrophoresed in the presence or absence of reducing agents, and immunoblotted with the same antibody. Size of protein markers (in kDa) is shown on left. (**B**) TOXCAT analysis of EBC5-16 oligomerization. The transmembrane domain of TC2-3 or EBC5-16 was inserted into the maltose binding protein/ToxR fusion protein and expressed in *E. coli* containing a ToxR-dependent chloramphenicol acetyl transferase (CAT) gene. CAT activity was measured *in vitro* after normalizing for the amount of fusion protein in the extract. Wild-type GpA and the dimerization-defective GpA G83I mutant were used as controls, and results are normalized to CAT activity induced by the GpA transmembrane domain. Graph shows the average of five independent experiments, each done in triplicate. Error bars represent standard error of the mean. A student t-test determined that the difference between EBC5-16 and TC2-3 samples was statistically significant, p < 10^-5^. (**C**) The sequences of EBC5-16 and EBC5-16-CCSS (amino acids 12 to the C-terminus) are shown, with position 25 and the cysteine to serine mutations in red. EBC5-16 (solid line) and EBC5-16-CCSS (dashed line) were expressed in BaF3/HA-hEPOR cells from the MSCV-puro vector. After puromycin selection, viable cells were counted on the indicated days after growth factor removal.

A fraction of TC2-3 forms a disulfide bond-linked homodimer mediated by the cysteines at the C-terminus of the protein [7]. To determine if EBC5-16 also forms a homodimer, cell extracts were prepared from BaF3/HA-hEPOR cells expressing either EBC5-16 or TC2-3, and replicate samples were immunoprecipitated with αE5, which recognizes the fixed C-terminus of TC2-3 and EBC5-16. One set of the samples was then treated with reducing agents to disrupt disulfide bonds, and the other set was left untreated. Samples were then electrophoresed in the presence of SDS to dissociate non-covalent dimers and immunoblotted with αE5. As shown in Figure 5A, under reducing conditions, TC2-3 and EBC5-16 migrated with similar mobility indicative of a monomer. Under non-reducing conditions, in addition to the monomeric form, a slower migrating band with mobility expected for a dimer was observed for both proteins, indicating that EBC5-16, like TC2-3, forms a disulfide bond-linked homodimer. Strikingly, a significantly higher fraction of EBC5-16 forms a dimer than TC2-3. Because there is only a single amino acid difference between the two proteins, this increase in homodimerization is due to the serine residue at position 25. The finding that a large fraction of EBC5-16 forms a disulfide bond-linked homodimer implies that, like the E5 protein, it adopts a type II transmembrane orientation, placing the C-terminus (containing the cysteines) in the non-reducing extracellular or luminal space. If EBC5-16 interacts directly with the hEPOR, this orientation would align EBC5-16 in an anti-parallel fashion relative to the transmembrane domain of the hEPOR, a type I transmembrane protein.

Cammett *et al.* used a TOXCAT assay to show that the central hydrophobic segment of TC2-3 can act as a transmembrane domain and undergo non-covalent oligomerization in bacterial membranes [7]. In this assay, the transmembrane domain to be tested is linked to the monomeric transactivation domain of ToxR, an oligomerization-dependent transcription factor. The level of ToxR-driven chloramphenicol acetyltransferase (CAT) expression as assessed by measurement of CAT activity is proportional to the strength of oligomer formation induced by the foreign transmembrane segment. To determine if EBC5-16 formed a stronger oligomer than TC2-3, we performed a TOXCAT assay with the transmembrane domains of EBC5-16 and TC2-3 (amino acids 8 to 32, lacking the C-terminal cysteines) inserted into ToxR. As shown in Figure 5B, the transmembrane domains of TC2-3 and EBC5-16 induced higher CAT activity than the transmembrane domain of the positive control, GpA, indicating that both traptamers form non-covalent oligomers in this system. Notably, EBC5-16 induced a statistically-significant 50% increase in CAT activity compared to TC2-3, indicating that the transmembrane domain of EBC5-16 forms a stronger oligomer than TC2-3. This finding corroborates the biochemical results that a higher fraction of EBC5-16 is present as a dimer in murine cells.

The results presented above demonstrated that EBC5-16 displays increased dimerization compared to TC2-3. To assess the importance of dimerization in EBC5-16 activity, we mutated both cysteines in the C-terminus of EBC5-16 to serine (to generate EBC5-16-CCSS). This mutant was expressed in BaF3/HA-hEPOR cells, and growth factor independence was assessed. As shown in Figure 5C, EBC5-16-CCSS did not confer growth factor independence, demonstrating that the cysteines, and presumably dimerization, are necessary for EBC5-16 activity. Taken together, these results raised the possibility that the increased activity of EBC5-16 is due to increased dimerization.

### Mapping the homodimer interface of EBC5-16

**Figure 6:**
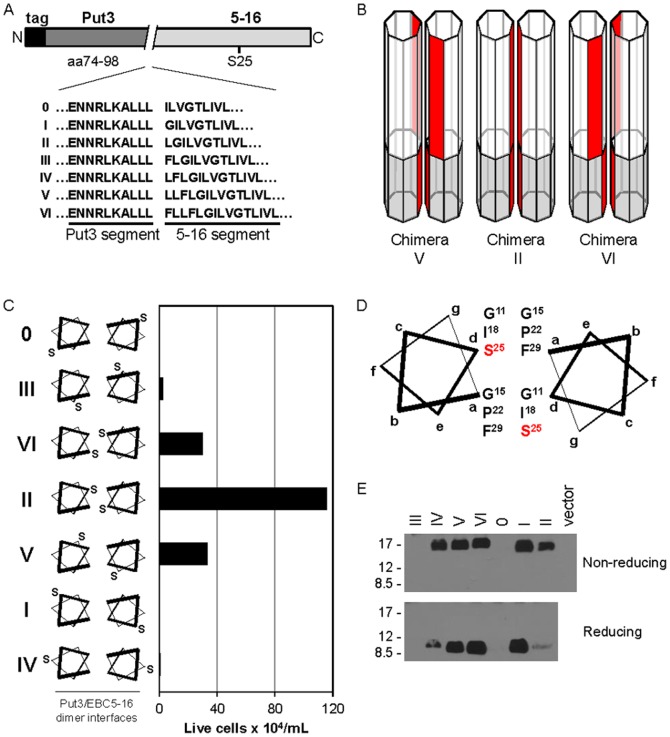
Mapping the EBC5-16 homodimer interface with Put3 fusion proteins. (**A**) Schematic diagram of the fusion proteins constructed between the dimerization domain of Put3 and the transmembrane domain of EBC5-16. The sequences show the point of fusion for each of the chimeras. The different points of fusion cause the relative positions of the amino acids of the EBC5-16 segment to rotate relative to the fixed interface of the Put3 segment. Because inserting seven residues would rotate the EBC5-16 segment by two full turns (720°), inserting a single amino acid would rotate each helix by 103°. Therefore, inserting three or four residues at the point of fusion will rotate the helices by 309° or 412°, respectively, generating structures in which the orientation of the helices is most similar to the original structure. Thus, in the series of seven consecutive insertion constructs, the interfaces can be placed in the following order in terms of their similarity: 0, III, VI, II, V, I, IV, as is listed in panel C. (**B**) Heptagonal prisms representing α-helical monomers within Put3/EBC5-6 dimers V, II, and VI. The Put3 and EBC5-16 segments are shaded in gray and white, respectively. The dimer interfaces of native Put3 and EBC5-16 are shaded in red. (**C**) (Left) helical wheel diagrams of the seven Put3/EBC5-16 dimers, with Ser25 shown for orientation, are shown. (Right) BaF3/HA-hEPOR cells expressing these chimeras from the RVY-puro vector were tested for their ability to proliferate in the absence of growth factors. Viable cells were counted six days after growth factor removal. Graph shows results of a representative experiment. Similar results were obtained in three independent experiments. (**D**) Helical wheel diagram of the predicted EBC5-16 dimer (from Put3/EBC5-16 chimera II), with interface residues shown. Ser25 is highlighted in red. (**E**) Extracts were prepared from BaF3/HA-hEPOR cells expressing empty RVY-puro vector or a Put3/EBC5-16 chimera from RVY-puro, immunoprecipitated with αE5, separated in the presence or absence of reducing agents, and immunoblotted with an anti-AU1 antibody. Size of protein markers (in kDa) is shown on left.

To determine which amino acids constitute the homodimer interface of EBC5-16, we used an approach we developed to identify the dimer interface of the BPV E5 oncoprotein, which was subsequently confirmed by biophysical studies [12], [17]. We constructed a set of plasmids encoding fusion proteins in which EBC5-16 was fused at seven consecutive residues to the dimerization domain of the yeast transcription factor, Put3, containing an N-terminal AU1 epitope tag (Fig. 6A). This segment of Put3 contains a leucine zipper motif that forms a left-handed coiled-coil homodimer, which will in essence force the fused protein of interest into a left-handed coiled-coil, whose interface residues can be predicted from the known structure of the Put3 dimer and the point of fusion [18], [19]. By fusing the Put3 segment at sequential residues of EBC5-16, each of the seven possible left-handed coiled-coil helical registers of the dimeric EBC5-16 segment is generated (schematic diagrams of representative chimeric protein dimers and helical wheel diagrams of all of them are shown in Figs. 6B and 6C, respectively). The residues that constitute the homodimer interface of native EBC5-16 can be inferred from the fusion protein that displays the highest biological activity.

Each of the Put3/EBC5-16 chimeras was cloned into the pRVY-puro vector and used to infect BaF3/HA-hEPOR cells. After cells were selected with puromycin for expression of the chimera, growth factors were removed from the medium, and viable cells were counted. As shown in Figure 6C, only construct II conferred robust growth factor independence. Chimeras V and VI were also active, but at a lower level than chimera II, whereas the other chimeras were inactive. Similar results were obtained if the chimeras were expressed from MSCV (data not shown). Strikingly, the three active chimeras are predicted to generate related structures, in which the orientation of the EBC5-16 segments differs by one register, with the most active structure (chimera II) flanked by the two less active ones (Fig. 6C). We conclude that the structure adopted by chimera II reflects the orientation of the native EBC5-16 homodimer.

Based on the known interface of Put3 and the point of fusion with the EBC5-16 segment, we predicted the residues forming the interface of the chimera II dimer (and by inference of EBC5-16 itself) are Gly11, Gly15, Ile18, Pro22, Ser25, and Phe29, as illustrated in the helical wheel diagram in Figure 6D. Thus, Ser25, which is responsible for the increased activity of wild-type EBC5-16 compared to TC2-3, is predicted to be in the homodimer interface. In addition, Gly11 and Gly15 lie in the predicted interface and constitute a glycine-x-x-x-glycine motif (GxxxG motif, where x can be any amino acid). This motif is frequently found in the interface of transmembrane domain homodimers [20]–[23].

To determine whether the Put3/EBC5-16 fusion proteins were expressed and dimeric, extracts were prepared from cells transduced with the Put3 chimeras, immunoprecipitated with αE5, and either treated with reducing agents or left untreated. Samples were then electrophoresed in the presence of SDS and immunoblotted with an anti-AU1 antibody. As shown in Figure 6E, in the presence of reducing agents, chimera II is expressed at a low level, despite being the most active chimera. Thus, the high-level activity of chimera II is not a result of increased expression compared to the other chimeras. Furthermore, two of the three chimeras with little or no activity (I and IV) were highly expressed, indicating that the inactivity of these constructs was not due to lack of expression. However, chimeras 0 and III were expressed at very low levels, possibly because of reduced stability, so their biological activity cannot be assessed. In the absence of reducing agents, all of the detectable fusion proteins migrated, as expected, as dimers, due to the presence of the heterologous Put3 dimerization domain and the C-terminal cysteines.

### Mutational analysis of the homodimer interface

**Figure 7:**
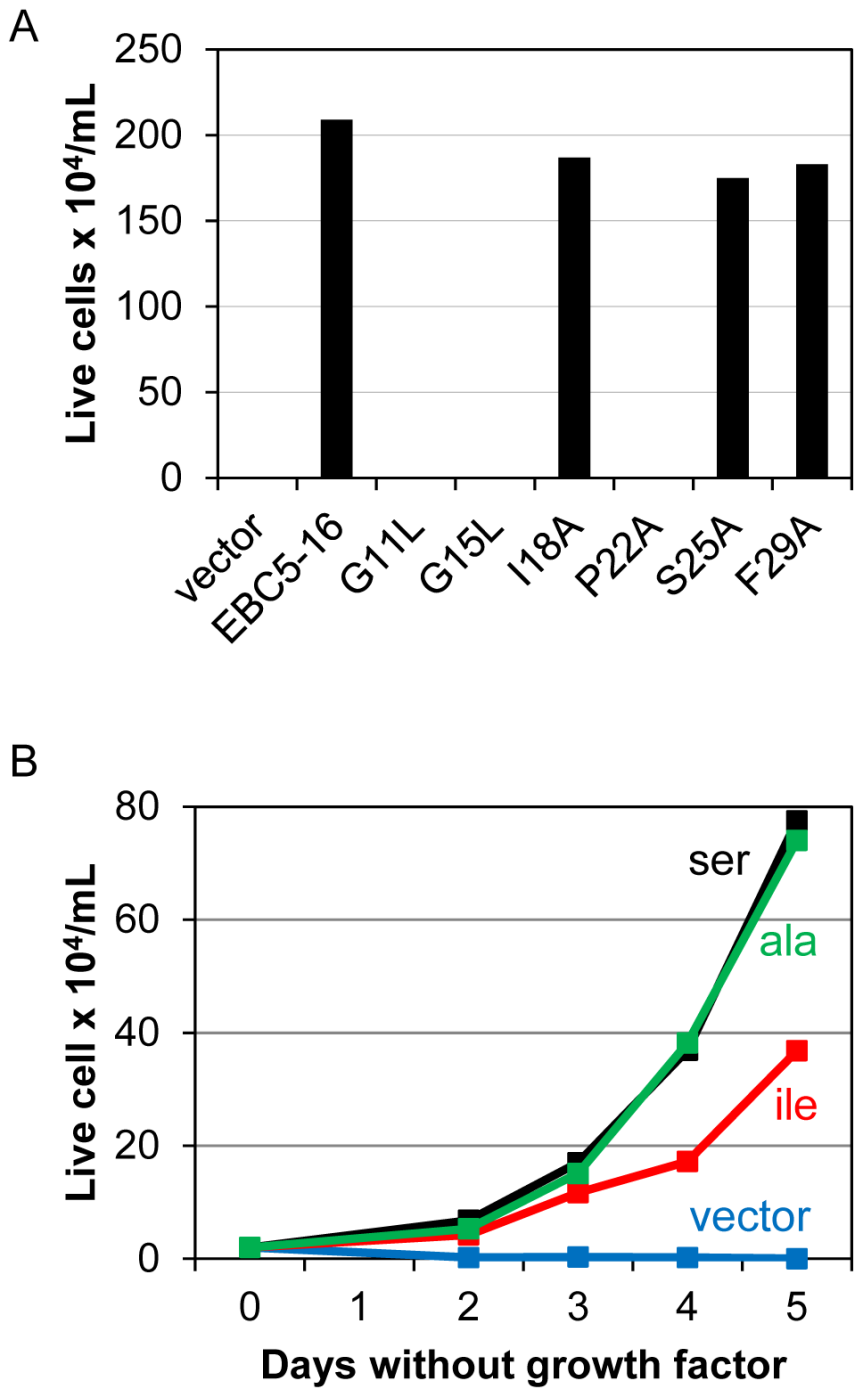
Mutational analysis of EBC5-16. (**A**) BaF3/HA-hEPOR cells expressing EBC5-16 or the indicated point mutant from MSCV-puro were tested for their ability to proliferate in the absence of growth factors. Viable cells were counted four days after growth factor removal. Graph shows the results of a representative experiment. Similar results were obtained in three independent experiments. (**B**) BaF3/hEPOR cells expressing empty RVY-hygro vector (blue), TC2-3 (red), EBC5-16 (black), or EBC5-16 S25A point mutant (green) were tested for their ability to proliferate in the absence of growth factors. The amino acid at position 25 is shown, according to the same color code. Viable cells were counted on the indicated days. Two and a half percent heat-inactivated FBS was used instead of 10%.

We constructed point mutations at each of the predicted interface positions in EBC5-16 to test whether these residues are essential for activity. BaF3/HA-hEPOR cells were infected with MSCV retrovirus expressing each of these mutants. After selection with puromycin, growth factors were removed from the medium, and viable cells were counted. As shown in Figure 7A, mutation of Gly11, Gly15, or Pro22 to leucine or alanine eliminated the ability of EBC5-16 to confer growth factor independence, demonstrating that these residues are required for the activity of EBC5-16, whereas the other mutants, including S25A, were active. These results indicated that three of the putative interface residues, Gly11, Gly15, and Pro22, are individually required for biological activity.

The ability of the S25A mutant to confer growth factor independence suggested that the serine did not form interhelical hydrogen bonds to allow activity, because the alanine side-chain cannot hydrogen bond. Similarly, the original activator, TC2-3, contains an isoleucine at position 25, which also lacks the ability to hydrogen bond. To directly compare the activities of the proteins with different amino acid substitutions at position 25, we infected BaF3/hEPOR cells with RVY retrovirus expressing EBC5-16, EBC5-16 S25A, or TC2-3 (which differ only by serine, alanine, and isoleucine, respectively, at position 25), selected for infected cells, and removed the growth factors from the medium. As shown in Fig. 7B, the S25A mutant conferred similar activity to that of EBC5-16, both of which were more active than TC2-3, further indicating that intermolecular hydrogen bonding is not required for activity and that smaller residues are better tolerated than a large, bulky hydrophobic residue at position 25.

### Molecular modeling indicates the predicted homodimer interface is energetically plausible

**Figure 8:**
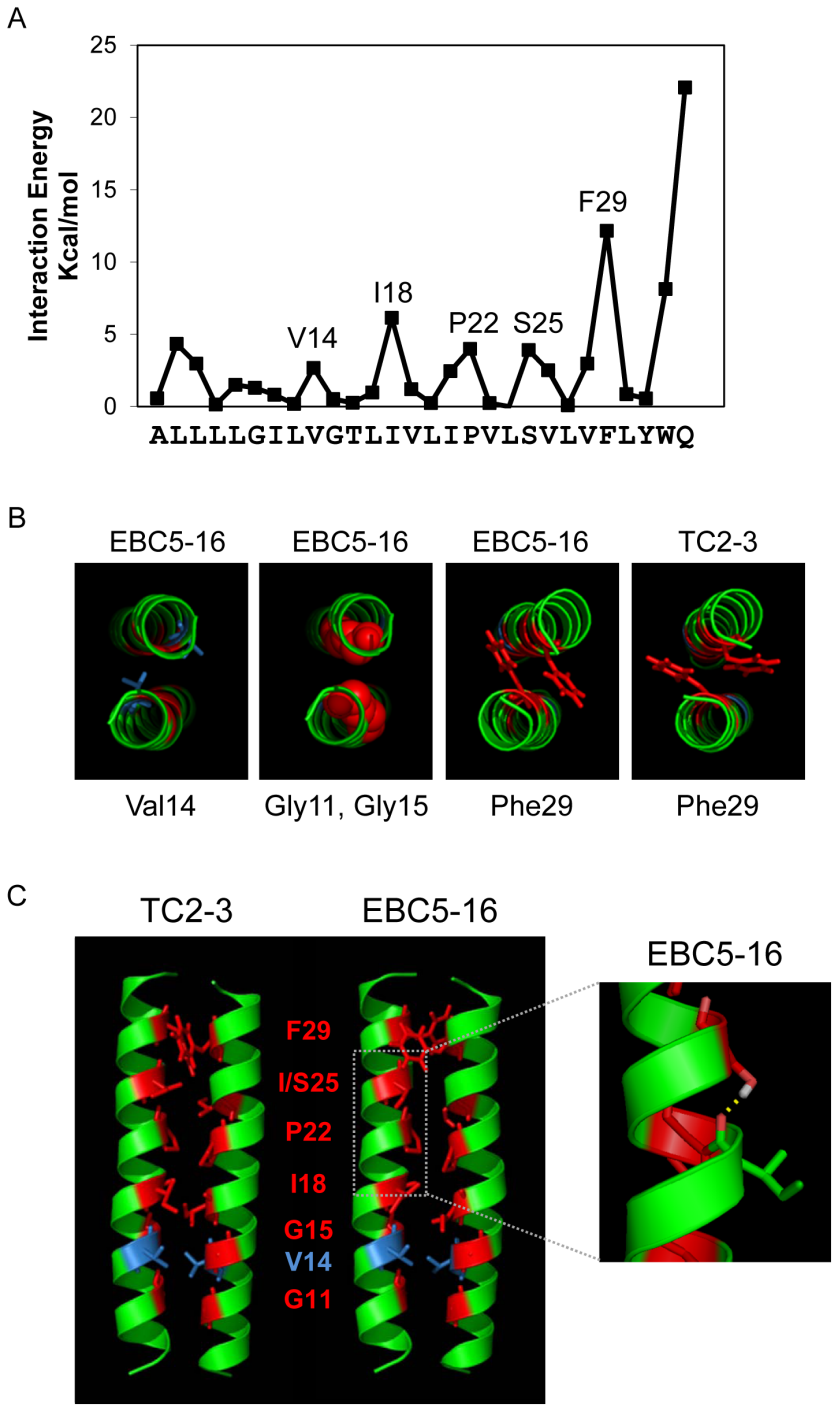
Molecular modeling of TC2-3 and EBC5-16. (**A**) The graph shows the interhelical interaction energy of the amino acids in the EBC5-16 homodimer CHI model discussed in the text. The sequence used for the modeling is shown at the bottom. (**B**) Axial views of helical backbone of the CHI models. First panel shows the view from N-terminus with Val14 side-chain shown in stick figure. Second panel shows view from N-terminus with Gly11 and Gly15 shown as space-filling. The right two panels show the view from C-terminus of EBC5-16 and TC2-3, with Phe29 side-chain shown in stick figure. (**C**) Left panel: Models of the TC2-3 and EBC5-16 homodimers predicted by CHI simulation, shown in lateral ribbon view with interface residues predicted by the Put3 experiments in red and Val14 in blue. Right panel: Zoomed-in view of an EBC5-16 monomer showing intramolecular H-bonding of the Ser25 side-chain to the backbone carbonyl group of Ile21, represented by the dotted yellow line. Oxygen atoms are shown in pink, hydrogen atoms are shown in white.

We used molecular modeling to determine whether the homodimer interface assigned by the Put3 experiments was energetically plausible and to explore the contribution of Ser25 to homodimer formation. The CHI molecular dynamics simulation and energy minimization protocol was used to generate structural models of the EBC5-16 homodimer. This technique was used previously by us and others to study homodimerization of the E5 protein and other transmembrane protein activators of the PDGFβR [2], [4], [6], [24], [25]. The structural calculations were performed based on the active Put3 chimera II, using the last four residues of Put3 up to the point of fusion (Ala95 through Leu98) fused to Leu10 to Gln33 of EBC5-16. Six different symmetric, left-handed coiled-coil, low energy clusters were obtained, one of which predicted Ser25 to be in the interface. The plot of the interaction energies of this model shows the energetic contribution of each residue to the stability of the homodimer interface (Fig. 8A). Importantly, this CHI model is consistent with the interface inferred from the Put3 experiments, in that Ile18, Pro22, Ser25, and Phe29 all lie in the homodimer interface in this model and contribute to the interaction energy of the dimer. The two interfacial glycines predicted by the Put3 experiments (Gly11 and Gly15) did not appear in the CHI interaction energy plot because glycine lacks a side-chain and thus cannot contribute directly to the energy of the dimer. Therefore, the glycines in the GxxxG motif most likely stabilize the dimer by allowing each monomer of EBC5-16 to approach one another more closely and pack more tightly, as has been observed frequently in other homodimeric transmembrane domains, including the GpA transmembrane dimer [26]. Consistent with this view, inspection of the CHI model revealed that Gly11 and Gly15 are at or near the dimer interface of EBC5-16, as is Val14, which makes a minor contribution to the interaction energy (Figs. 8A and 8B, left two panels).

There are two additional noteworthy observations from the modeling. First, comparison of the models for EBC5-16 and TC2-3 showed marked re-arrangement of the amino acid side-chains within the interface as a consequence of the isoleucine to serine mutation (Figs. 8B, right two panels, and 8C). This was most dramatic in the case of the Phe29 side-chains, where the aromatic rings are oriented differently in the models of the TC2-3 and the EBC5-16 dimer (Fig. 8B, right two panels). Second, Ser25 did not appear to form a hydrogen bond across the helical interface, but rather hydrogen bonds with the main chain carbonyl of Ile21 on the same helix (Fig. 8C inset), consistent with the mutational data shown in Figure 7 that a hydrogen-bonding side-chain at position 25 is not required for activity.

### Reconstitution of the homodimer interface

**Figure 9:**
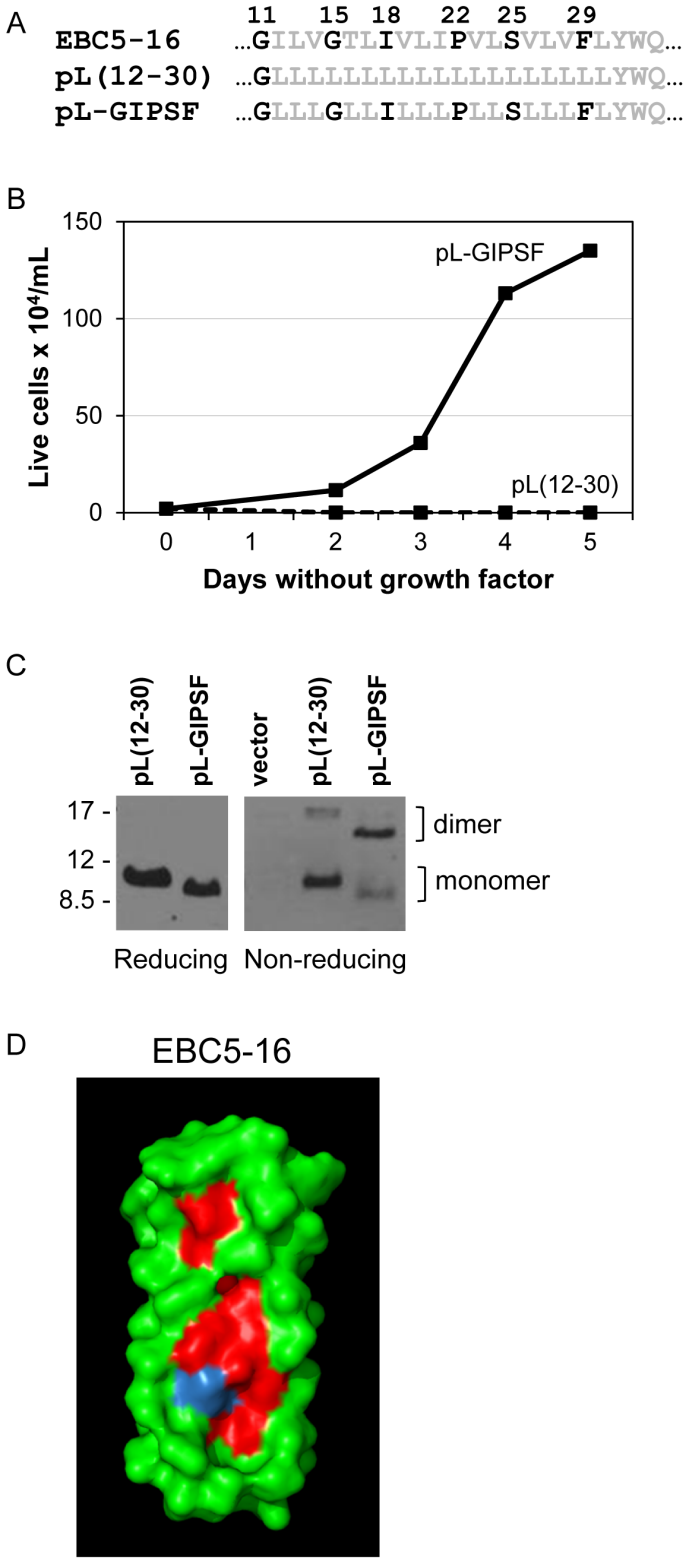
Residues predicted to be in the homodimer interface of EBC5-16 are sufficient to restore dimerization and activity. (A) Amino acid sequences of the transmembrane domains of EBC5-16, the pL(12-30) poly-leucine construct, and pL(12–30) with predicted interface residues (in black) added back (pL-GIPSF). (B) BaF3/HA-hEPOR cells expressing pL(12–30) (dashed line) or pL-GIPSF (solid line) from MSCV-puro were tested for their ability to proliferate in the absence of growth factors. Viable cells were counted on the indicated days after growth factor removal. Graph shows the results of a representative experiment. Similar results were obtained in four independent experiments. (C) Extracts were prepared from BaF3/HA-hEPOR cells expressing AU1-tagged pL(12–30) or pL-GIPSF from MSCV-puro, immunoprecipitated with αE5, electrophoresed in the presence or absence of reducing agents, and immunoblotted with the same antibody. Size of protein markers (in kDa) is shown on left. Gel was soaked in 200 mM DTT for 30 min prior to transfer. (D) Surface representation of the EBC5-16 dimer with the predicted interface residues in red and Val14 in blue.

To explicitly test the role of the predicted interface residues in activity, we inserted them into an inactive, monomeric construct containing poly-leucine in place of the transmembrane domain (residues 12–30) of EBC5-16 (this construct is designated pL(12–30)) and determined whether these residues were sufficient for homodimerization and activity. Gly11 was present in both constructs because it was present in the fixed backbone of pL(12–30) (Fig. 9A). The five remaining predicted interfacial amino acids, namely Gly15, Ile18, Pro22, Ser25, and Phe29, were inserted into pL(12–30) to generate pL-GIPSF. BaF3/HA-hEPOR cells were infected with retrovirus expressing AU1-tagged pL(12–30) or the add-back construct. After selection with puromycin, growth factors were removed from the medium and viable cells were counted over time. Although pL(12–30) was inactive, cells expressing pL-GIPSF, the construct containing the interface predicted by the Put3 model, conferred growth factor independence (Fig. 9B), demonstrating that the predicted interface residues are sufficient to confer biological activity.

To determine if the interface residues were sufficient for dimerization, cell extracts were prepared from BaF3/HA-hEPOR cells expressing pL(12–30) and the interface add-back construct. The samples were then immunoprecipitated with αE5, subjected to SDS-PAGE under reducing and non-reducing conditions, and immunoblotted with αE5. As shown in Figure 9C, in the presence of reducing agents, both constructs were expressed at similar levels, demonstrating that the inactivity of pL(12-30) was not due to poor expression. In the absence of reducing agents, pL(12–30) migrated primarily as a monomer, while the add-back construct migrated primarily as a dimer. This result demonstrated that the predicted interface residues, Gly15, Ile18, Pro22, Ser25, Phe29, restoring the GxxxG motif, are sufficient in a poly-leucine context for homodimer formation and biological activity.

## Discussion

Protein engineering and directed evolution are powerful approaches to design, optimize, and analyze biologically active proteins. In previous work, we isolated an artificial, dimeric, 44-amino acid transmembrane protein, TC2-3, which activates the hEPOR and supports erythroid differentiation of primary hHPCs in the absence of EPO, even though it bears no sequence similarity to EPO [7]. However, TC2-3 is much less active than EPO in inducing erythroid differentiation. To examine the basis for hEPOR activation by transmembrane proteins as well as to gain a better understanding of the structure of hEPOR traptamers, we isolated and characterized a more active version of TC2-3. By subjecting a library of TC2-3 mutants to more stringent selection conditions, we isolated a mutant, EBC5-16, which differs from TC2-3 by only a single amino acid but supports erythroid differentiation with activity comparable to EPO, as assessed by cell-surface GpA expression. Like TC2-3, EBC5-16 is dimeric, can serve as a transmembrane domain, and functionally interacts with the transmembrane domain of the hEPOR. The high activity of EBC5-16 in inducing erythroid differentiation is particularly striking because it is so dissimilar to EPO, which is monomeric, soluble, and binds the extracellular domain of the EPOR. We used a similar directed evolution strategy to optimize traptamers that down-regulate CCR5 [8]. These results demonstrate the utility of random mutagenesis and selection to optimize artificial transmembrane domains that target single-pass and multi-pass transmembrane proteins.

Several lines of evidence suggest that the enhanced activity of EBC5-16 is due to increased homodimerization caused by the substitution of a serine for an isoleucine. First, EBC5-16 exists in cells as a disulfide bond-linked homodimer. Second, mutation of the cysteines that mediate covalent dimerization abolishes activity. Third, the serine substitution increases the fraction of EBC5-16 in the dimeric form, as assessed by non-reducing gel electrophoresis and TOXCAT experiments. Although the TOXCAT result indicated the transmembrane domain of EBC5-16 is sufficient for dimerization in bacterial membranes, the defect caused by the cysteine mutations implies that in mammalian cells the dimer is stabilized by disulfide bonds. Similarly, the transmembrane domain of BPV E5 lacking the C-terminal cysteines has intrinsic dimerization potential, but the presence of the cysteines or fusion to a heterologous dimerization domain is required for high-level activity in mammalian cells [12], [24], [27]. Finally, we identified Gly11, Gly15, Ile18, Pro22, Ser25, and Phe29 as the residues constituting the homodimer interface of EBC5-16. Importantly, insertion of these interfacial residues into an inactive variant of EBC5-16 containing a monomeric poly-leucine transmembrane domain was sufficient to reconstitute a dimeric protein that activates the hEPOR. Although our results show unequivocally that dimerization of EBC5-16 is required for activity, it remains possible that alterations in amino acid side-chain orientation caused by the I25S substitution has a direct effect on the increased activity of EBC5-16 compared to TC2-3.

The identification of the homodimer interface provides insight into the nature of the interactions that stabilize the EBC5-16 dimer. Transmembrane helix homodimerization is typically mediated by van der Waals interactions and various types of hydrogen bonds [28]-[30]. Although Ser25 lies in the homodimer interface of EBC5-16 and its side-chain has hydrogen bonding potential, it does not appear to increase dimerization of EBC5-16 via interhelical hydrogen bonding. Substitution of the Ser25 to alanine, which cannot hydrogen bond, does not affect the activity of EBC5-16. Furthermore, in the preferred model of the EBC5-16 homodimer, the serine side-chain hydrogen bonds with the polypeptide backbone on the same helix. Thus, the small side-chains of serine and alanine at position 25 appear to allow the helices to approach one another more closely and form more favorable packing contacts. In contrast, replacement of serine with several large hydrophilic amino acids capable of hydrogen bonding abolished activity (unpublished results). We also note that the orientation of several of the other side-chains in the interface is markedly different in the EBC5-16 model compared to TC2-3. This side-chain rearrangement may also contribute to more optimal packing of the helices and the formation of additional van der Waals contacts that stabilize the dimer. Similarly, in other systems, van der Waals interactions can make a significant contribution to the tight packing of transmembrane dimers, and conservative amino acid substitutions at such tightly-packed positions can affect the ability of a transmembrane protein to dimerize [31]–[34].

Two glycine residues and the proline are predicted to lie in the EBC5-16 homodimer interface and are required for EBC5-16 activity. Although glycine and proline can be helix-disrupting in soluble proteins [35], [36], this does not appear to be the case for EBC5-16. Glycine is readily accommodated in helices in hydrophobic environments [28], [36]–[39]. Notably, a GxxxG motif is present in >30% of all transmembrane domains and facilitates dimerization by permitting the close approach of transmembrane helices, providing a relatively flat surface for tight interhelical packing interactions and allowing larger neighboring side-chains to participate in favorable van der Waals interactions [20]–[23], [28], [40]. β-branched residues adjacent to these glycine residues in GxxxG motifs, such as isoleucine, valine, and threonine, are also important for homodimerization of transmembrane helices, including the GpA transmembrane domain [21], [32], [41]. Three out of the four residues flanking the glycines in EBC5-16 are β-branched, suggesting that this motif plays a similar role in dimer formation by EBC5-16 and GpA. Prolines are also often present in the middle of transmembrane domains [42]–[47]. Because of its rigidity and the absence of a backbone amine hydrogen bond donor, proline can induce a kink in transmembrane sequences, which can allow a conformational change that leads to transmission of a downstream signal [42], [43], [48]–[50]. Similarly, Pro22 in the middle of EBC5-16 is essential for activity, and the molecular modeling suggested that it induces a small kink in EBC5-16. The presence of an essential GxxxG packing motif in the homodimer interface and the requirement for a small interfacial amino acid at position 25 for maximal activity provides further support for the hypothesis that tight packing of the EBC5-16 dimer is crucial for its increased activity.

In addition to forming a homodimer, EBC5-16 must contain amino acids that mediate activation of the EPOR. The hEPOR is primarily a pre-formed dimer in its inactive state [51]–[53], and a conformational change or rotation of the receptor molecules appears to activate the EPOR in response to EPO binding or genetic manipulations that force the EPOR monomers to adopt a particular orientation [54]–[56]. We hypothesize that EBC5-16 induces a similar structural change in the hEPOR, likely through binding directly to the transmembrane domain of the receptor (unpublished results). Strikingly, addition of the predicted EBC5-16 interface residues to an inactive poly-leucine construct was sufficient not only for homodimerization but also for activity, demonstrating that these residues restored a functional interaction with the hEPOR. Six leucine residues in the pL-GIPSF are also present in EBC5-16 itself and might interact with the receptor or with another protein that mediates hEPOR activation. Alternatively, one or more of the predicted interface residues may participate in not only homodimer formation but also the interactions required for receptor activation. The surface representation of the CHI model indicates that portions of the interfacial side-chains are accessible at the surface of the dimer for such heteromeric interactions (Fig. 9D). Our identification of the EBC5-16 homodimer interface provides the foundation for further mechanistic studies and allows us to better understand how these small transmembrane proteins function and interact with their target.

In comparison to TC2-3, EBC5-16 supports growth factor independence at lower expression levels and is more effective at inducing erythroid differentiation. The enhanced dimerization of EBC5-16 presumably increases its ability to activate the hEPOR or causes a quantitative or qualitative change in signaling output. However, the levels of tyrosine phosphorylation of the hEPOR, JAK2, and STAT5 were similar in cells expressing EBC5-16 and TC2-3. We hypothesize that TC2-3 and EBC5-16 induce an as-yet-unidentified difference in EPOR signaling, for example, by affecting which specific tyrosines are phosphorylated. Similarly, different orientations of the EPOR intracellular domains can result in qualitatively different signaling outcomes [55]. It is also possible that the signaling output of the EPOR in response to EBC5-16 differs in some regards from the output of EPO-stimulated receptor. In fact, EBC5-16 stimulates some aspects of erythroid differentiation, such as GpA expression, better than others, suggesting that EPOR-mediated erythoid differentiation is not an all-or-nothing process. Further analysis of EPOR signaling in response to various activators may reveal new aspects of EPOR action.

As well as illuminating aspects of transmembrane protein interactions and cell physiology, our results may have practical implications. Transmembrane domains derived from native proteins have been added to cells as peptides or expressed as short proteins, resulting in their incorporation into cell membranes and biological activity [57]–[61]. In fact, hydrophobic peptides derived from a naturally-occurring transmembrane domain can localize to appropriate tissues after systemic injection into animals [62], [63]. Our results indicate that artificial transmembrane proteins may also be the source of biologically active hydrophobic peptides, which may have important research and even clinical uses. Similarly, genes encoding small, cell-autonomous, transmembrane proteins may find use in *ex vivo* gene therapy. In fact, artificial transmembrane domains may have more favorable properties than proteins derived from natural sequences. For example, traptamers can display high specificity, such as the ability to distinguish between human and mouse EPOR. Increased specificity or signaling differences of artificial transmembrane domains compared to natural ligands may reduce harmful side effects, including those described following administration of high doses of EPO to patients [64]–[68]. The utility of these approaches obviously depends on the specificity of traptamers toward a wide range of cellular proteins, which has not yet been assessed, and on the development of methods to properly deliver these agents and regulate their expression or activity. Nevertheless, our results suggest that biologically active transmembrane proteins can serve as templates for new classes of potent peptide or peptidomimetic agents that modulate a wide array of cellular and viral transmembrane proteins.

## Supporting Information

Table S1 **Oligonucleotides used in library, clone, and mutant construction; recovery of inserts from selected cells; and measurement of RNA levels.**
(DOCX)
