# Supplementary material for: Mapping the Homodimer Interface of an Optimized, Artificial, Transmembrane Protein Activator of the Human Erythropoietin Receptor
Source: PLoS One. 2014 Apr 30;9(4):e95593. doi: 10.1371/journal.pone.0095593 (PMC4005772; doi:10.1371/journal.pone.0095593)
Supplement: Table S1 — Oligonucleotides used in library, clone, and mutant construction; recovery of inserts from selected cells; and measurement of RNA levels. (DOCX) [file pone.0095593.s001.docx]

| **Library construction and recovery** | | |
| --- | --- | --- |
| TC2-3 mutagenized library | 1F | 5′-AGTGTCCTAGGAEXJJXZZXJZZJEJJJXJEXJZXJJXJEXJJJJZXJJXJEXJZ  XZJXJZXJXXJJXZTACTGGCAGCATTTCGAATGCTCCTG-3′  **E** A:C:G:T ratio, 1:0.02:0.02:0.02; **J** A:C:G:T ratio, 0.02:1:0.02:0.02; **X** A:C:G:T ratio, 0.02:0.02:1:0.02; **Z** A:C:G:T ratio, 0.02:0.02:0.02:1 |
|  | 1R | 5’-GCAGCTCGAGGATCCTTAAAAGGGCAGACCTGTACAGGAGCATTC  GAAATG-3′ |
| Amplification | 2F | 5′-CGCAAGTGTCCTAGGA-3′ |
|  | 2R | 5′-GCAGCTCGAGGATCC-3′ |
| Rescue | 3F | 5’-TCCTCCCTTTATCCAGCCCTCAC-3’ |
|  | 3R | 5’-CCCTAACTGACACACATTCCACAG-3’ |

| **RT-PCR** | | |
| --- | --- | --- |
| β-globin | 4F | 5′-GGTGGTGAGGCCCTGGGCAGG-3′ |
|  | 4R | 5′-GGCCATCACTAAAGGCACCG-3′ |
| GAPDH | 5F | 5′-CTGCACCACCAACTGCTTAG-3′ |
|  | 5R | 5′-GTCTTCTGGGTGGCAGTGAT-3′ |

| **Codon-optimization of EBC5-16** | | |
| --- | --- | --- |
| EBC5-16opt | 6F | 5’-GCTCGAGACCATGGCCAACCTGTGGTTCCTGCTGTTCCTGGGCATC  CTGGTGGGCACCCTGATCGTGCTGATCCCCGTGCTGAGCGTGCTGGTGTTCCTGTACTGGCAGCACTTCGAGTGCTCCTG-3’ |
|  | 6R | 5’-GCACGAGCTGGATCCTCAGAAGGGCAGGCCGGTGCAGGAGCACTC  GAAGTG-3’ |
| Amplification | 7F | 5’-CGCTCGAGACCATGG-3’ |
|  | 7R | 5’-GCACGAGCTGGATCC-3’ |

| **Construction of TOXCAT chimeras** | | |
| --- | --- | --- |
| pccKAN | 8F | 5’-gctacgctagcttgttcctaggaattctggtcgg-3’ |
|  | 8R | 5’-gctacggatcccccagtacaggaagacgagcacg-3’ |

| **Construction of pL(12-30)** | | |
| --- | --- | --- |
| N-terminal  AU1 tag | 9F | 5’-TCGAGCAATTGACCATGGCCGACACCTACAGGTACATCAACCTGT  GGTTCCTGCTGTTC-3’ |
|  | 9R | 5’-CTAGGAACAGCAGGAACCACAGGTTGATGTACCTGTAGGTGTCGG  CCATGGTCAATTGC-3’ |
| pL(12-30) | 10F | 5’-gcatgcctaggcctgctgctgctgctgctgctgctgctgctgctgc  tgctgctgctgctgctgctgctgtactggcagcacttcgagtgctcc-3’ |
|  | 6R | Same as 6R |

| **Mutagenesis of EBC5-16** | | |
| --- | --- | --- |
| CC🡪SS | 11F | 5′-CGAATCCTCGTCGACAGGTCTGCCCTTTTAAG-3′ |
|  | 11R | 5′-GATCCTTAAAAGGGCAGACCTGTCGACGAGGATT-3′ |
| G11L  codon optimized | 12F | 5’-GTTCCTGCTGTTCCTGCTCATCCTGGTGGGCAC-3’ |
|  | 12R | 5’-GTGCCCACCAGGATGAGCAGGAACAGCAGGAAC-3’ |
| G15L  codon optimized | 13F | 5’-tgggcatcctggtgctcaccctgatcgtgc-3’ |
|  | 13R | 5’-gcacgatcagggtgagcaccaggatgccca-3’ |
| I18A  codon optimized | 14F | 5’-GGTGGGCACCCTGGCCGTGCTGATCCC-3’ |
|  | 14R | 5’-GGGATCAGCACGGCCAGGGTGCCCACC -3’ |
| P22A  codon optimized | 15F | 5’-GATCGTGCTGATCGCCGTGCTGAGCGTG-3’ |
|  | 15R | 5’-CACGCTCAGCACGGCGATCAGCACGATC-3’ |
| S25A  codon optimized | 16F | 5’-GATCCCCGTGCTGGCCGTGCTGGTGTTCC-3’ |
|  | 16R | 5’-GGAACACCAGCACGGCCAGCACGGGGATC-3’ |
| S25A | 17F | 5’-tcatccccgtcctcgccgtgctcgtcttcc-3’ |
|  | 17R | 5’-ggaagacgagcacggcgaggacggggatga-3’ |
| F29A  codon optimized | 18F | 5’-GAGCGTGCTGGTGGCCCTGTACTGGCAGC-3’ |
|  | 18R | 5’-GCTGCCAGTACAGGGCCACCAGCACGCTC-3’ |
